# Iron-Fortified Drinking Water Studies for the Prevention of Children's Anemia in Developing Countries

**DOI:** 10.1155/2011/815194

**Published:** 2011-08-04

**Authors:** Jose E. Dutra-de-Oliveira, J. Sergio Marchini, Joel Lamounier, Carlos A. N. Almeida

**Affiliations:** ^1^Department of Internal Medicine, Ribeirão Preto School of Medicine, University of São Paulo, Av Bandeirantes 3900, 14049-900 Ribeirao Preto, SP, Brazil; ^2^Department of Pediatrics, Medical School Belo Horizonte, Av Professor Alfredo Balena 190, 30130-100 Belo Horizonte, MG, Brazil; ^3^Department of Pediatrics, Ribeirão Preto School of Medicine, University of São Paulo, Av Costábile Romano 2201, 14096-900 Ribeirao Preto, SP, Brazil

## Abstract

Anemia and iron deficiency should receive special attention considering their high prevalence and serious consequences. For prevention, globally it is recommended to increase dietary iron intake, iron fortification of industrialized foods, and medical iron supplementation. Food fortification for the prevention of iron deficiency in developing countries should consider carriers locally available and consumed daily, requiring limited infrastructure and technology. Drinking water is the iron carrier we have been working for years for the prevention of iron deficiency and anemia in small children in Brazil. It was shown that studies with iron-fortified drinking water were proved to be effective on children's anemia prevention. Water is found everywhere, consumed daily by everyone may be easily fortified with simple technology, is low priced and was effective on the prevention of children's anemia. Fortification of drinking water with iron was locally implemented with the direct participation of the government and community. Government authorities, health personnel and population were part of the project and responsible for its community implementation. The mayor/municipality permitted and supported the proposal to supply it to children at their day-care centers. To keep the children drinking water iron fortified supply an officially authorized legislation was also approved.

## 1. Introduction

Iron deficiency and iron anemia are prevalent worldwide, affecting a large proportion of global population. It has been estimated the worldwide incidence of anemia to be 47% in children under five, 30% in no pregnant women of childbearing age, and 42% in pregnant women [1]. It has been identified as the most prevalent micronutrient deficiency in the world [2]. It is mostly present in developing world and is said to affect over 2 billion people. Infants, preschool children, and pregnant women are the main groups at risk for iron deficiency and iron anemia. Its prevention includes food with high-iron bioavailability and a nutritious daily diet. Alternative solutions involve selective plant breeding or genetic engineering that will increase iron content or reduce absorption inhibitors in dietary staples, iron enrichment of industrialized foods, and iron supplementation with pharmacological medicine products. 

Food fortification has been regarded as the safest and most effective means to supplement diets with low-iron content. Industrialization required for preparation of iron food fortification has often been a problem requiring technology not available in less developed countries. Frequently, poor regions do not have the necessary infrastructure or even adequate local for processing food fortification. Or even in these poor and less developed countries or regions they do not produce or use international agencies food recommended to be fortified. Several foods have been used as nutrient carriers, with their own particularities. Important considerations are that international food-based suggestions for food fortification require changes in local diet habits and even food importation. Fortification of foods should include locally available and consumed foods. Another serious problem for prevention of iron deficiency through food fortification in developing and less developed countries as wheat flour, for example, is to have it available everywhere and consumed daily in amounts that could be sufficient to supply their daily need of iron. Small children can only eat small amounts of bread and infants have problems to eat solid foods. In Brazil where iron-fortified wheat flour was made mandatory by law, preliminary results have shown that under five children anemia did not get better [3]. Reality has shown that in spite of all available knowledge, new technologies, supplementation, and fortification programs, the problem of iron deficiency and iron anemia is kept as one of the greatest public health nutrition problems in less and developing world. 

At the Micronutrient Forum held at Turkey in 2007 [4] several papers reported again the magnitude and severity of the world anemia problem. It was shown that the problem is far from being solved, especially in less developed countries and poor areas of the world. On the other hand it is also to be said that several studies on iron food fortification for the prevention of anemia keep going in several parts of the world. In Mexico [5], fortification of rice with iron using Ultra Rice technology was shown to be an efficacious strategy for preventing iron deficiency. In Guatemala [6] fortification of sugar with FeNaEDTA improved the iron status of the semirural population. In Vietnam [7] the use of NaFeEDTA fortified salt was shown effective to control iron deficiency in child bearing women. There are also a large number of other experimental studies using a variety of carriers, as iron-fortified soy sauce in Indonesia, fish sauce in Cambodia, and brown bread in South Africa. Mandatory flour fortification with iron and folic acid and of maize is going on in some countries, such as Pakistan, where the prevalence of anemia is quite high [8]. 

It has to be clear that a fortified food for anemia prevention in developing countries to be effective should use carriers locally available and legally implemented at the community. Certainly one such alternative is our proposed utilization of drinking water as an iron carrier. Water by itself is an essential liquid nutrient and to it could be dissolved other nutrients as iron and other minerals and vitamins. WHO held a meeting on water on health and published a report on the subject [9]. Water is available and used daily everywhere by everyone from infants at birth to old age, which does not happen with various other nutrient food carriers. An important characteristic of water as a carrier is that it is liquid, proper to be given to infants, children, and sick people. The bioavailability of nutrients carried in water increases with their solubility. Fortification of drinking water with iron is a simple process, and solutions may be prepared locally starting with a concentrate water iron sulfate solution at a drug medicine store or similar set-up, local always available even in small towns. A concentrated iron solution is bottled in 1 L pot and sent to the day-care centers where a diluted solution is offered to the children. At the day-care a calculated amount of the concentrated iron solution is diluted to large drinking water bottles of 20 litters. We have worked with a final concentration of 10 to 20 mg/L of iron from ferrous sulfate, which by the usual daily children water intake was shown to be sufficient for the control of the children's anemia. This ferrous sulfate iron-fortified water shows a light yellowish color and has a small metallic taste. Starting the utilization with low amount of iron, the children get used to the fortified water with no problems. 

Quite a few iron salts were tested by us and some of them like the NaFeEDTA did not change the color of the water, does not change its taste, and is biologically active. This salt is a little more expensive than ferrous sulfate. Our studies have also worked with different ways through which iron-fortified water could be supplied to the population in a large community. One of them was adding iron to the city water distribution system. This is the way through which fluoride has been carried out in several places in the world to prevent tooth decay; we thought to use it for the prevention of anemia at community level. Different iron salts as pills and tablets to be used at homes were also tested to supply extra iron to all members of a family, including one with a sparkling formulation with iron plus ascorbic acid. The fact is that water iron fortification solutions may be prepared locally and used for cooking and drinking water, supplying iron to all members of families or institutions. Drinking water iron fortified could also be used to increase the daily iron intake of pregnant and lactating women. An additional and important advantage of liquid iron water solutions over other kinds of solid food iron carriers as we appointed is that a liquid can be easily given to small infants and children, the largest iron deficient and anemia age groups. Recent data from Brazil has shown that 72.5% of infants 6 to 12 months of age were found to be iron deficient and anemic in a surrey in the Northwestern of Brazil [10]. 

Data on our previous experimental, animal, children, and community on the use of drinking water as an iron carrier and strategies for its implementation at community levels in Brazil will be presented and commented (Table 1).

## 2. Iron from Complex Salts and Its Bioavailability in Rats [11]

The objective of these laboratory experiments was to show the effect of adding different iron salts to drinking water and checking their effects on color, turbidity, and taste, through physical and chemical tests. Ferric ammonium citrate, ferric chloride, ferric gluconate, ferric hydroxide, ferric sulfate, and ferric nitrate, diluted in water, were tested. Iron citrate, chloride, gluconate, and ferrous sulfate produced small color changes at 1 mg/L and 5 mg/L concentrations, when measured initially and after seven days. Tests with iron sodium EDTA added to the water solution showed practically no color change and no metallic taste; the solution remained clear and transparent. The presence of chloride in iron-fortified water increased color and turbidity, though it had no effect on NaFeEDTA solution. Along with the physicochemical tests, acceptance trials of these different water-fortified solutions were run continuously with over 500 children. Different from what many expected, our children rapidly adapted to the low metallic taste of ferrous sulfate. 

It was also found that iron-fortified water turbidity decreases with the addition of ascorbic acid or citric acid to the solution. Water solutions of Fe gluconate, citrate, and sulfate had less color and turbidity than those of polimaltosed OHFe³ and Fe³ nitrate. An anemic weanling rat test was carried out where a group of rats did not get iron in their drinking water and the other group receive water with ferrous sulfate, NaFeEDTA, iron-glycine chelate, and complex ferric orthophosphate. The control animals became anemic after 35 days, 9.1 g/dL of Hb, and the ones receiving ferrous sulfate, NaFeEdta, and Fe glycine chelate 13.0–13.9 g of Hb. The animals drinking iron Fe orthophosphate had 10.6 g Hb/dL. Weight gain, hemoglobin, hematocrit, transferring saturation, iron hemoglobin, and relative iron biological value were checked in all animals. NaFeEDTA and iron aminochelate produced similar results as ferrous sulfate. Orthophosphate iron had lower biological value. 

## 3. Drinking Water as an Iron Carrier to Control Anemia in Preschool Children in a Day-Care Center [16]

In this study, we look at the effect of Fe-fortified drinking water offered to 31 preschool children under 5, during eight months. The children attended a day-care institution in the city of Ribeirão Preto, starting early in the morning and leaving around 5 pm in the afternoon. They received three meals a day and had free access to iron-fortified water. Iron sulfate solution was added to the drinking water, at a concentration of 20 mg of elemental iron per liter. Clinical and anthropometric data were obtained for each child. Blood was collected and hemoglobin and serum ferritin measured before, four and eight months after the intervention began. Anemia decreased significantly after the introduction of iron-fortified drinking water (ferrous sulfate only). The number of Fe-deficient children decreased drastically after they started drinking the Fe-enriched water. Mean hemoglobin values increased from 10.6 to 13.7 g/dL and serum ferritin from 13.7 to 25.6 mcg/L, after eight months. There were no problems related to the ferrous sulfate addition or the acceptance of drinking the Fe-enriched water. It was concluded that the iron-enriched water is a practical alternative to supply iron and prevent anemia. 

## 4. Iron Fortification of Domestic Drinking Water to Prevent Anemia among Low Socioeconomic Families in Brazil [17]

The objective of this study was to evaluate through blood parameters (hemoglobin and ferritin levels) the effect of an iron-fortified drinking water used in the preparation of family meals. Participants were families of small children with anemia who were previously seen at our University Hospital in the City of Ribeirão Preto. They had borderline blood anemia iron parameters. Twenty-one families of low socioeconomic status including 88 subjects participated in the project and were divided into two groups. Twelve families, 22 fathers and/or mothers, and 22 children less than 6 years old with hemoglobin levels ≤11 g/dL participate in the study. Daily intakes of selected nutrients were measured. A solution of water iron + ascorbic acid (AA) in small bottles was given to a group of 12 families mothers to be added to their cooking pots (concentration 10 mg of Fe and 60 mg AA/L). Nine other families (18 fathers and mothers and 26 similar children) were supplied with the same type of water, but without added iron or ascorbic acid, during four months. Blood samples were collected at the beginning and at the end of the trial to measure Hb and ferritin levels at the end in both children and adults. The group receiving the iron + ascorbic acid-fortified water increased their Hb and ferritin blood values and the control maintained concentrations similar to initial levels. It was concluded from this study that iron + ascorbic acid fortification can be add at home for their home food cooking and is effective to increase blood hemoglobin and ferritin in adults and children. It can be a simple and effective way to supply iron to low socioeconomic families where the iron intake may be found to be low.

## 5. Effect of Fortification of Drinking Water with Iron Plus Ascorbic Acid or with Ascorbic Acid Alone on Hemoglobin Values and Anthropometric Indicators in Preschool Children in Day-Care Centers in Monte Alto, Southeast Brazil [15]

Ascorbic acid is known to increase iron absorption. We ran a trial to look at the effect of ascorbic acid on iron utilization. This study was conducted with 150 children in six day-care centers of the village of Monte Alto. Their diet included approximately 10 mg of iron, mainly from vegetable sources and low levels of vitamin C (28% of daily requirements for the age group). Half of the children received iron plus 100 mg of AA in the water and the other half received only the 100 mg AA, with no added iron, during a six-month period. The prevalence of anemia (Hb ≤ 11 mg/dL) was 45% for the iron plus AA group and 31% for the AA group, at the beginning of the experiment, dropping to 31.7 and 17.1%, respectively, at the end of the study. This led us to the conclusion that the day-care diet already supplied some iron because there was found an anemia decrease in the children receiving only the day-care diet and that ascorbic acid alone increases its availability. We had also previously shown that fortification of orange juice with iron (8 mg a day) offered to preschool children during four months also increased significantly their iron status measured by hemoglobin levels [18].

## 6. Implementation of Iron Fortification Programs at Community Level

An important aspect of our studies, along with the demonstration that iron carried out by drinking water could control iron deficiency anemia in infants and small children, was planned in a way that the program should implanted locally as part of the local government children assistance program. The community-applied research studies on the use of drinking water as an iron carrier were always previously approved by the University Research Committee and the County Community Authorities where they were to be carried. The project always started with our close contact with the municipality mayor and public health authorities, linked to county children assistance programs. There were explained to them details of the project that would start with a clinical nutrition check-up of all children attending their day-care centers. We would check locally their blood for anemia and all children would receive a fortified drinking water during 6 to 8 months, when a new clinical and blood test would be repeated. It was explained that we would supervise, train, and work with local people who already work at the center, like teachers, care takers, and helpers for them to know what to do in relation to the anemia project. The local healthy community personnel, physicians, nurses, dietitians, helpers, children, mothers, and educators were involved. Several meetings were carried out to explain the project, to look for the presence of anemia in the children, and to tell them on the importance of iron on health, growth, learning, and development of the children. The results were always shown to the mothers, day-care helpers, and local health authorities. The mayor was to have the compromise to support the research project and at the end of it to send to the city council a law project that would require mandatory addition and availability of iron water fortification to all children at the municipality day-care centers. A booklet on anemia printed with topics on food, nutrition, and anemia was distributed at the community. 

## 7. Conclusions

This review has shown the following.

- Anemia is a hidden deficiency, present and out of control problem in Brazil as it is in several other parts of the world. It is affecting millions of persons, particularly infants, young children, future generations, and women.
- World food fortification programs are going for more than 20–30 years concentrating efforts on the prevention of iron deficiency. Results are far from what it would be expected or desirable. This is an unacceptable situation.
- A recent 2010 anemia prevalence data in a poor area of the Northeast Brazil, a children survey 6 to 60 months showed 45% occurrence of anemia. The highest incidence 75.2% was on the 60- to 12-month age-group [10].
- Strategies on iron food fortification to control childhood anemia in Brazil have been recently reviewed. Iron food fortification for the control of childhood anemia in Brazil includes mandatory fortification of milled wheat and corn flour, fortification of milk, iron fortification of biscuits and bread rolls, and iron fortification of potable drinking water [3].
- Our Group at the Medical School of Ribeirao Preto, University of Sao Paulo have been working since 1994 with iron fortification of drinking water for small children [16]. It has all the perspectives to be a better worldwide carrier than other more traditional wheat flour, corn flour, soya, and so forth.
- Iron fortification of drinking water has been shown to be efficient for the prevention of children's iron deficiency and anemia and easily implemented at community level. Water is consumed everywhere by everyone; infants drink water, can be iron fortified locally, and is quite cheap. Public level and mandatory legal law approach showed iron fortification drinking water to be feasible for local public implementation.

## Figures and Tables

**Table 1:** Clinical work carried out in preschool children with iron fortified drink water in Brazil.

Reference	Age group (years)	Number of Participants	Place	Fortification compounds	Indicator of ID g/dL		Duration
					Before	After	
Dutra-de-Oliveira et al. [12]	2–6	31	Daycare Center of Ribeirão Preto Brazil	Ferrous sulfate (20 mg Fe/L)	Hb: 10.6 ± 1.1 FT: 13.7 ± 8.9	Hb: 13.0 ± 1.1 FT: 25.6 ± 10.5	8 months

Dutra-de-Oliveira et al. [13]	Families Adults + Children 1–6	88	Homes of Ribeirão Preto Brazil	Ferrous sulfate 10 mg/L + L-ascorbic acid 100 mg/L	Children: Hb: 10.9 ± 1.1 FT: 27.6 ± 21.6	Children: Hb: 11.7 ± 1.1 FT: 33.8 ± 22.1	4 months
					Adults: Hb: 12.9 ± 1.7 FT: 74.8 ± 41.3	Adults: Hb: 13.7 ± 1.7 FT: 106.2 ± 93.9

Lamounier et al. [14]	Children 1/2–6	321	Daycare Center Belo Horizonte Brazil	Ferrous sulfate 10 mg/L + L-ascorbic acid 100 mg/L	Hb: 11.8 ± 1.3	Hb: 12.9 ± 1.4	5 months

Almeida et al. [15]	1–6	74	Daycare Center of Monte Alto Brazil	Ferrous sulfate (10 mg Fe/L)	Hb: 11.3 ± 1.3	Hb: 11.5 ± 1.3	6 months
